# Opportunities to Target the Life Cycle of Epstein-Barr Virus (EBV) in EBV-Associated Lymphoproliferative Disorders

**DOI:** 10.3389/fonc.2019.00127

**Published:** 2019-03-15

**Authors:** James P. Dugan, Carrie B. Coleman, Bradley Haverkos

**Affiliations:** ^1^Division of Hematology, University of Colorado, Aurora, CO, United States; ^2^Division of Immunology, University of Colorado, Aurora, CO, United States

**Keywords:** EBV (Epstein-Barr virus), EBV-associated cancers, Lymphoproliferative disease (LPD), EBV latency, viral life cycle inhibitors

## Abstract

Many lymphoproliferative disorders (LPDs) are considered “EBV associated” based on detection of the virus in tumor tissue. EBV drives proliferation of LPDs via expression of the viral latent genes and many pre-clinical and clinical studies have shown EBV-associated LPDs can be treated by exploiting the viral life cycle. After a brief review of EBV virology and the natural life cycle within a host we will discuss the importance of the viral gene programs expressed during specific viral phases, as well as within immunocompetent vs. immunocompromised hosts and corresponding EBV-associated LPDs. We will then review established and emerging treatment approaches for EBV-associated LPDs based on EBV gene expression programs. Patients with EBV-associated LPDs can have a poor performance status, multiple comorbidities, and/or are immunocompromised from organ transplantation, autoimmune disease, or other congenital or acquired immunodeficiency making them poor candidates to receive intensive cytotoxic chemotherapy. With the emergence of EBV-directed therapy there is hope that we can devise more effective therapies that confer milder toxicity.

## Introduction

Epstein-Barr Virus (EBV), also called human herpesvirus 4 (HHV-4), is a lymphotropic gamma-herpes virus that infects >90% of adults worldwide (1). EBV is defined by a discrete viral life cycle with primary infection, latency, and lytic reactivation phases (2). There are two peaks of EBV infection as measured by seroconversion, age 2–4 years and 15 years (3). In children the primary infection may go undetected or present as an upper respiratory infection. In adults the symptoms of primary infection can be more severe, leading to a syndrome known as infectious mononucleosis. After primary infection the virus remains dormant in latency with memory B cells serving as the primary reservoir for persistence (4). For the vast majority of individuals latent EBV infection does not seem to have any serious health consequences. However, dysregulation of latency or inability to control lytic infection can lead to the development of lymphoproliferative diseases (LPDs) and lymphoma.

EBV was originally discovered in the context of African endemic Burkitt lymphoma (5, 6) and is classified as a Class I carcinogen by the International Agency for Cancer Research (7). EBV-associated malignancies are well characterized in individuals with suppressed immune systems such as after solid organ transplantation or in the setting of HIV/AIDS but are also recognized in patients without overt immunodeficiencies (8, 9). EBV is associated with nearly all nasopharyngeal carcinoma (NPC), approximately 10% of gastric carcinomas, 30–40% of classical Hodgkin lymphoma, a subset of diffuse large B-cell lymphoma (DLBCL), and other T/NK cell LPDs (8, 10–13). After primary infection EBV latency is defined by distinct gene expression programs (14–16). Viral latency is mediated through promoter silencing, characterized by limited protein expression, and categorized by four latency types (latency 0-III) (17–19). Expanded knowledge around the manipulation of DNA methylation and histone acetylation has led to a better understanding of the virus' ability to facilitate viral persistence in healthy individuals (20, 21), alter transcription factor accessibility (22, 23), silence tumor suppressor genes (24–26), and ultimately potentiate tumor development and growth (27–29). In the first part of our review we will discuss the regulation of the latent and lytic phases of EBV. In the second part we will show how researchers have capitalized on these mechanisms to target EBV and treat associated malignancy. EBV may prove to be the Achilles' heel of EBV-associated tumorigenesis and targeting the viral life cycle may help patients avoid toxic chemotherapeutics and receive more tailored and effective therapy.

## Regulation of EBV Gene Expression

EBV latency is defined by a restricted, but variable protein expression that is specific to the host cell type (e.g., lymphoid or epithelial) or tumor origin. *In vitro*, B-cell immortalization is mediated by the viral latency III program in which all six EBV nuclear antigens (EBNAs 1, 2, 3A, 3B, 3C, and –LP), three latent membrane proteins (LMPs 1, 2A, and 2B), and viral non-coding RNAs (EBERs, miRNAs, and BARTs) are expressed and lead to the establishment of lymphoblastoid cell lines (1). The latency III program is the least restrictive latency type and is seen in LPDs associated with immunosuppression such as AIDS-associated DLBCL and post-transplant lymphoproliferative diseases (PTLDs) (30). Latency II is defined by expression of EBNA1, LMP1, and LMP2A/B and is most closely associated with Hodgkin lymphoma, NPC, and T/NK cell lymphomas (31–34). Only EBNA1 is expressed during latency I, which is associated with Burkitt lymphoma, as well as gastric carcinoma (35, 36). Latency 0 refers to the persistence of the viral genome in the absence of viral gene expression, which is associated with non-dividing memory B-cells (37).

The EBNA family of genes is among the viral genes differentially regulated by epigenetic modification during EBV latency. The six EBNA gene products expressed during the latency III program are constructed from one extensively spliced latency transcript. The EBV latency C promoter (Cp) is the origin for transcription of the EBNA latency proteins (38). CpG methylation of Cp plays an important role in regulating viral latency and limiting viral gene expression in normal lymphocytes and in certain malignancies including Burkitt, Hodgkin, AIDS associated DLBCL, and NK cell lymphomas, as well as NPC (39–42). During latent phase within the host cell the EBV genome is maintained as a circular episome that undergoes replication once per cycle, initiating from a region called oriP. EBNA1 is the only viral protein required to replicate from oriP and segregate the EBV episomal genomes in latency (43). EBNA1 has been shown to be responsible for promoting and maintaining latency (44). Compared to other EBV gene products EBNA1 is poorly recognized by CD8+ T-lymphocytes (45). The down regulation of the more immunogenic EBNA antigens (EBNA2, 3A-C), LMP1, early lytic antigens, and lytic viral kinases contributes to the virus' ability to evade the immune system during latency (46).

EBNA2 is critical for the transformation of B-cells (47) and is directly responsible for the initiation of transcription of EBV proteins associated with latency III like LMP1 and LMP2A/B (1, 48). EBNA2 is implicated in the Notch pathway, which contributes to viral latency by downregulating LMP1 and preventing the expression of BZLF1 (49). EBNA2 has been seen to target the c-Myc oncogene, which is important for EBV-induced B-cell immortalization *in vitro* (50). The EBNA3 family of proteins are also involved in B-cell transformation and essential for EBV persistence (51). EBNA2 and EBNA3 work together to regulate the expression of cellular and viral gene expression (52). EBNA3 may have a direct impact on progression through the cell cycle disrupting G2/M checkpoint (53) and has been shown to interact directly with human histone deacetylases influencing epigenetic regulation (54, 55). The EBNA family of proteins have been shown to work together in concert with host cellular machinery to affect histone acetylation and DNA methylation, directly impacting transcription of EBV related proteins to maintain latency (56–59).

LMP 1 and LMP 2A/2B are found in latency II and latency III EBV infected cells. LMP1 is essential for B lymphocyte growth transformation and for the survival of EBV transformed B-cells (60). LMP1 mimics CD40 signaling, which is a key B-cell costimulatory receptor (61). LMP1 behaves as a prototypical oncogene *in vitro* and is associated with upregulation of antiapoptotic proteins (62, 63) and stimulation of cytokine production (64). Specifically, constitutive activation of NF-kB and mitogen-activated protein kinase (MAPK) are supported by LMP1 and critical to lymphoblastoid cell line survival (65, 66). Knockdown of LMP1 downregulates NF-kB signaling and induces apoptosis (67). Expression of LMP1 in transgenic mice induces the development of B-cell lymphomas (68). LMP2A/B support LMP1 functions, as well as suppress B-cell receptor signaling (69). The inhibition of B-cell receptor signaling regulates EBV latency by preventing B-cell differentiation to plasma cells and effectively blocking the switch from latent to lytic replication (70). LMP1 and LMP2A signaling can induce expression of DNA methyltransferases (DNMT1, 3A, and 3B), which impacts major cellular pathway signaling. PARP1 mediates EBV replication during latency and LMP1 has been shown to alter expression of tumor-promoting genes by blocking histone methylation via PARP1 activation (71). LMP1 and LMP2A have been associated with hypermethylation and silencing of the PTEN gene in gastric carcinoma (72, 73). LMP1 induces the expression of the histone demethylase KDM6B, which has been associated with the pathogenesis of Hodgkin lymphoma (74). LMP2A is also implicated in the development of Hodgkin lymphoma via specific alterations in gene transcription (75). These examples highlight how EBV machinery can subvert the cell's normal epigenetic mechanisms thereby promoting viral latency and subsequent tumorigenesis.

EBV encodes many small non-coding RNAs (EBER1, EBER2, and viral miRNAs) that are widely expressed in infected cells (76, 77). Non-coding RNAs are expressed during all forms of EBV latency and also during the lytic cycle (78). Epigenetic manipulation by non-coding RNAs is thought to occur via recruitment of host transcription factors and chromatin regulators that modulate viral and host gene expression (79). Recruitment and thus alterations to host gene expression is mediated by viral RNA targeting of complementary sequences on cellular mRNA (80, 81). For example, EBER2 has been shown to target the B-cell transcription factor PAX5 via an RNA:RNA interaction (82). EBER1 has been shown to increase the expression of insulin growth factor-1 (IGF-1) and potentiate cellular proliferation in EBV associated gastric cancer (83). In fact, the EBERs, and in particular EBER1, have been shown to contribute to lymphoid hyperplasia and lymphoma on their own (84). There is evidence to suggest EBERs can increase IL-6 expression leading to the downstream activation of STAT3. This interaction may have a direct impact on host cell chemoresistance and migration (85).

The viral miRNAs are differentially expressed depending on the infected cell or tumor type. EBV miRNAs are involved with early B-cell proliferation and suppression of apoptosis (86, 87). The miRNAs are subdivided into two groups, Bam HI fragment H rightward open reading frame I microRNAs (BHRF1 miRNAs) and Bam HI-A rightward transcripts microRNAs (BART miRNAs), based on their locations (76, 88). The BARTs are a group of stable viral RNAs represented in every EBV infected cell type. Their expression is regulated by promoter methylation and treatment with a DNA methyltransferase increased the expression of BART miRNA transcripts (89). The BART promoter region is hypomethylated in NPC, which may explain why BART miRNAs are highly expressed in this tumor type (90, 91). Whether the BART miRNAs are translated to protein products remains controversial but is an important area of research for targeting EBV in malignancy (90, 92, 93). Expression of EBV miRNAs has been observed in gastric carcinoma (94), peripheral and cutaneous T-cell lymphoma (95–97), B-cell lymphoma cell lines, and NPC EBV-infected cells (76, 88) implicating EBV miRNAs in tumorigenesis.

When an EBV infected B-cell terminally differentiates to the plasma cell lineage the virus activates the lytic cycle genes and generates viral progeny (98). The switch from latency to the lytic cycles is mediated by two viral transactivator proteins, BZLF1 and BRLF1 (99). These two genes are influenced by DNA methylation of the viral genome. BZLF1 binds to methylated DNA and interacts with histone acetyltransferases to instigate expression of lytic promoters (100, 101). Expression of BZLF1 leads to a cascade of over 80 EBV gene products that results in viral replication and ultimately host-cell lysis. BRLF1 activates some early lytic genes through a direct binding mechanism (102, 103), while other gene activation is mediated through interactions with cellular transcription factors (104). BRLF1 activates phosphatidylinositol 3 kinase (PI3K), which is required for BRLF-mediated induction of lytic gene expression (105). During reactivation in an immunocompetent host, EBV-primed CD4+ and CD8+ memory T-cells can respond within hours and destroy virally infected cells before viral replication is finished (46). EBV expresses several gene products during lytic reactivation to directly counteract the T-cell response. Expression of BNLF2a, for example, prevents peptide presentation by MHC class I molecules through direct inhibition of antigen processing (106) and BGLF5, another lytic protein, augments the expression of MHC class I and II molecules (107). In addition, these lytic viral proteins can suppress pro-inflammatory cytokine release and temper the innate immune response including natural killer cell killing of EBV-infected B-cells (108).

This detailed knowledge of the viral life cycle provides opportunities to target features of the virus that promote lymphoproliferation. In the following section we will show how treatment strategies have capitalized on this to target EBV driven lymphoproliferation, thus providing novel treatment options. Ultimately though, we expect and look forward to future treatment approaches that will be more specific based on the improved understanding of the viral life cycle, including epigenetic modifications, outlined above.

## EBV Targeted Therapy

The various patterns of viral gene expression in LPDs has treatment ramifications (Table 1). For lymphomas like Burkitt and classical Hodgkin only a small subset of the latent gene profile is expressed offering limited and poorly immunogenic antigens to target. Alternatively, EBV-associated LPDs that arise as a result of immunosuppression generally express more gene products and are thus susceptible to antiviral directed therapy and reduction in immunosuppression, as is the case for EBV-associated PTLD after solid organ transplantation (109). In tumor types like EBV-associated PTLD or DLBCL with expanded viral protein motifs, constitutive activation of lytic proteins, such as viral thymidine kinases (vTKs) BXLF1 and BGLF4, has been demonstrated (110–113). Activation in vTKs result in phosphorylation of the nucleoside analogs ganciclovir (GCV), acyclovir, and zidovudine (AZT) (114–116). As a result, these antivirals can be effective in tumor types that demonstrate constitutive activation of lytic phase proteins; however, they are inactive against latent infection since there is no expression of the lytic kinases. There has been an evolving interest in developing techniques for inducing the lytic phase of the virus, sometimes referred to as the “kick and kill” strategy. In this scenario the virus is pushed into replicating so that phosphorylation of the nucleoside analogs can occur.

**Table 1:** Latent viral protein expression patterns in EBV-associated malignancy.

**Latency type**	**Associated diseases**	**EBV proteins expressed**		**LMP1**	**LMP2**	**EBER/miRNA**
		**EBNA1**	**EBNA2, 3A-C, LP**			
0	Burkitt Lymphoma	–	–	–	–	+
I	Burkitt Lymphoma	+	–	–	–	+
	Gastric carcinoma	+	–	–	(+)	+
II	Hodgkin's Lymphoma	+	–	+	+	+
	NPC	+	–	+	+	+
	T/NK LPD	+	–	+	+	+
	EBV+ DLBCL, NOS	+	(+)	+	(+)	+
III	AIDS associated DLBCL	+	+	+	+	+
	PTLD^*^	+	+	+	+	+

*DLBCL, diffuse large b-cell lymphoma; EBNA, EB viral nuclear antigen; EBV, Epstein-Barr virus; LMP, latent membrane protein; LPD, lymphoproliferative disorder; NPC, nasopharyngeal carcinoma; PTLD, post-transplant lymphoproliferative disorder*.

* *including lymphomatoid granulomatosis variant*.

*(), variable expression*.

The use of AZT and GCV in patients with immunosuppression-related EBV-associated B-cell lymphoma was originally investigated by the late Dr. William J. Harrington at the University of Miami in the 1990s, based on *in vitro* data showing that AZT and GCV additively induced apoptosis in EBV+ cell lines (117) and on anecdotal reports of disease regression in patients with HIV-associated lymphomas after exposure to AZT (118, 119). Harrington and colleagues reported rapid clinical responses in 4 of 5 patients using a regimen of intravenous AZT, GCV and interleukin (IL)-2 for 2–3 weeks, without any antineoplastic chemotherapy or radiation (117). This regimen was adopted by the AIDs Malignancy Consortium in a prospective study (AMC-019, NCT00006264) (120), and in 1998 a Phase II clinical trial of the AZT/GCV combination, based on the Harrington schedule, was opened for patients with primary CNS PTLD, a B-cell neoplasm that shares a number of clinical and biologic features with HIV-associated PCNSL, including near universal association with EBV, inconsistent response to immune restoration, and poor prognosis (121). The original Harrington regimen was amended to eliminate use of IL-2 in recipients of solid organ transplant and to include an extended 2-year maintenance phase or oral AZT and GCV following the initial intravenous 14-day “induction” phase. The Phase II trial was eventually closed due to difficulties with accrual associated in part with the rarity of the indication and with the severity and acuity of the target population.

Other strategies have been employed to induce the lytic phase of the EBV lifecycle and make the associated malignancies susceptible to antiviral therapy regardless of latency subtype. Preclinical studies have shown that pharmacologic induction with dexamethasone and rituximab induces lytic protein expression and renders EBV infected B-lymphocytes sensitive to ganciclovir (122). In a phase I/II study investigating EBV lytic induction in nasopharyngeal carcinoma, patients were given a combination of gemcitabine and valproic acid to induce lytic gene expression and then treated with valganciclovir. The authors were able to demonstrate safety of this regimen, as well as increases in EBV-DNA loads in the blood (123). Other chemotherapies like 5-fluororuacil, platinum agents, or paclitaxel in conjunction with antiviral medication have also induced lytic activation and increased sensitivity to antiviral therapy in NPC (124). Aspirin can induce lytic gene expression via suppression of NF-kB, which allows downregulation of LMP1 and subsequent expression of BZLF1. Treatment of EBV+ cells *in vitro* with aspirin and ganciclovir shows significantly improved cytotoxic effect than with either drug alone (125). In many cases investigators have been able to show synergistic effects between traditional chemotherapies and antiviral therapies, as well as increased levels of viral DNA in blood. Clinically, these patients seem to have transient and/or moderate side effects and improved quality of life, which are important benchmarks for a patient population whom would not have tolerated traditional high dose chemotherapy and radiation.

Exposure of latently infected B-cells *in vitro* to histone deacetylase inhibitors (HDACi) alters the promoter sequences or disrupts the gene silencing of BZLF1 and BRLF1 genes thereby inducing lytic reactivation (126). Support for this approach was first demonstrated in a lung transplant recipient with an EBV-associated immunoblastic lymphoma 4 months following transplantation (127). Based on work demonstrating that butyrate congeners could induce EBV lytic genes, including vTKs (128, 129), a cell line derived from the patient's tumor was exposed to arginine butyrate resulting in induction of EBV TK transcription. The combination of arginine butyrate and ganciclovir resulted in inhibition of cell proliferation and cell death. Arginine butyrate was added to the patient's existing treatment with ganciclovir with no apparent increase in toxicity. Though the patient succumbed to a systemic aspergillus infection that had preceded the administration of arginine butyrate therapy, pathologic examination of the tumor demonstrated substantial necrosis compared to pre-therapy histology (127). Additional support for the use of an HDAC inhibitor to sensitize EBV infected tumor cells to nucleoside antivirals was demonstrated in a phase I/II trial of arginine butyrate combined with ganciclovir in 15 patients with EBV-associated LPDs previously treated with chemotherapy and/or radiation (130). Arginine butyrate was administered by daily continuous IV infusion on an escalating dose schedule for 21 days of a 28-day treatment course, combined with a fixed dose of daily continuous ganciclovir. Eleven patients received at least 28 days of arginine butyrate and ganciclovir, and all 15 patients were evaluable for response. Significant antitumor activity was seen in 10 patients, with 4 complete responses (CRs) and 6 partial responses (PRs). Several HDAC inhibitors in addition to arginine butyrate can induce expression of EBV lytic phase genes *in vitro*, leading to the sensitization of EBV infected lymphoma cells to nucleoside antivirals (127, 131). Ongoing studies are evaluating the ability of HDAC inhibitors to sensitize EBV infected lymphoma cells, irrespective of latency subtype, to ganciclovir *in vivo* (NCT0339770).

As discussed, CpG promoter methylation is used by EBV to silence lytic phase genes and promote latency. CpG methylation can be reversed via pharmacologic demethylation promoting the expression of EBV lytic genes in latently infected cells. Drugs targeting this epigenetic mechanism include the DNA hypomethylators 5-azacitadine and decitabine. 5-azacitadine inhibits DNA methyltransferase, reduces methylation of CpG promoter regions, and activates transcription of EBNA2 (132). EBNA2 is directly associated with regulation of the latency III program and transcription of proteins LMP1 and LMP2A/B (1, 48) In one study, EBV promoter methylation was evaluated in patients with NPC and AIDS associated lymphomas before and after receiving 5-azacitadine. The authors were able to demonstrate demethylation to varying degrees in the latent and early lytic EBV promoters evaluated (133). Reducing CpG methylation and stimulating the more immunogenic EBV proteins may facilitate immune-mediated destruction of the tumor cells and make them sensitive to antiviral therapy. Ultimately, methylation of viral DNA may become a clinically useful tool to characterize the associated latency subtype and treat the corresponding associated LPD (134).

Lastly, we would like to discuss the role of adoptive T-cell immunotherapy. In a seropositive immunocompetent person EBV is actively monitored by EBV-specific cytotoxic T-lymphocytes (CTLs). In patients that are immunocompromised either via iatrogenic means as in the case of organ transplantation or via infectious means like HIV, EBV is permitted to proliferate due to the depletion of these CTLs. Adoptive T-cell immunotherapy involves infusing EBV-specific CTLs generated *in vitro* with the aim of reconstituting the EBV immunity and influence targeted destruction of EBV infected tumor cells. Importantly, native T-cells are HLA-restricted meaning a patient's T-cell will only recognize antigen presented by HLA molecules of their own allelic type. This has implications for sourcing EBV-specific CTLs. For example, in bone marrow transplant recipients PTLD is almost exclusively of donor origin, so attempts have been made to treat PTLD in this scenario with donor derived EBV-specific CTLs. Preparing non-specific populations of CTLs (i.e., donor lymphocyte infusions) can have serious consequences, such as graft vs. host disease, related to the alloreactivity of T-lymphocytes (135). Severe and even fatal graft vs. host disease have been seen after administration of non-specific allogeneic CTLs, which is why most new therapies use polyclonal EBV-specific HLA matched CTL lines prepared *in vitro* (136). In 1995 Rooney et al. introduced gene-modified EBV-specific T-lymphocytes to allotransplant recipients with EBV-associated LPDs. The authors showed that EBV-CTLs could generate a CR without associated infusional complications (137). In a total of 101 patients given EBV-CTLs for prophylaxis, none of the patients developed an EBV-associated LPD and 11/13 patients with an EBV-associated LPD had a CR (138). The majority of research on the clinical application of EBV-CTLs has focused on PTLD given the immunogenicity of these tumors and the full expression of EBV latency III antigens. In 2007 Bollard and colleagues showed that by increasing the frequency of LMP2-specific CTLs responses in immunocompetent patients were dramatically improved. They concluded that it was possible to generate immune responses to weak tumor antigens like LMP2 by *in vitro* manipulation of CTL antigen recognition (139). In a 2014 study Bollard et al. looked at LMP1/2 specific EBV-CTLs in patients with a variety of latency II associated malignancy including Hodgkin lymphoma, DLBCL, T/NK cell lymphomas, and NPC. Twenty-nine of 50 patients treated with autologous CTLs in remission from high-risk or multiple-relapsed disease had an EFS of 82% and 11 of 21 patients treated with active disease experienced a CR (140). In a another trial, patients with extranodal NK cell lymphoma were given LMP1/2a specific CTLs and 9 of 10 patients showed a sustained remission (141). There are multiple active trials evaluating EBV-specific and EBV-antigen specific CTLs for use in patients with EBV-associated LPDs (see Table 2 for a list of trials).

**Table 2:** Clinical trials targeting EBV in malignancy.

**Category**	**Intervention**	**Drug class**	**Indication**	**Phase**	**Status**	**Identifier**
Lytic Induction	VRx-3996 Valganciclovir	HDACi Antiviral	R/R EBV+ lymphomas	1b/2	Recruiting	NCT03397706
	Doxorubicin + MTX Zidovudine + Hydroxyurea	Cytotoxic Antiviral Antimetabolite	R/R EBV+ lymphomas PTLD	II	Completed	NCT01964755
	LBH589 RAD001	HDACi mTOR inhibitor	NPC, EBV+ lymphomas, any EBV+ solid tumor	1b/2	Active, not recruiting	NCT01341834
Epigenetic manipulation	Vorinostat Azacitadine	HDACi Hypomethylator	NPC, Extranodal nasal NK/T cell lymphoma	1	Active, not recruiting	NCT00336063
CTL	GRALE CTL	LMP, BARF1, EBNA1	EBV+ Lymphomas T/NK LPD CAEBV	1	Recruiting	NCT01555892
	LMP1/2 CTL	LMP1/2	EBV+ Lymphomas T/NK LPD CAEBV Leiomysarcoma	1	Recruiting	NCT01956084
	MABEL CTL	LMP, BARF-1, EBNA1	EBV+ Lymphomas T/NK LPD CAEBV	1	Recruiting	NCT02287311
	PBTLs and EBV-CTLs	CD19-CD28 EBV-specific	R/R low or int grade B-cell lymphoma or B-CLL	1	Active, not recruiting	NCT00709033
	CMD-003	EBV-specific	EBV+ Extranodal NK/T-cell lymphoma	2	Recruiting	NCT01948180

*CAEBV, chronic active epstein-barr virus; CTL, cytotoxic T-lymphocyte; EBV, Epstein-Barr virus; HDACi, histone deacetylase inhibitor; LPD, lymphoproliferative disease; MTX, methotrexate; NPC, nasopharyngeal caricinoma; R/R, relpase/refractory*.

Another T-cell mediated strategy that has shown some promise in EBV-mediated malignancy involves checkpoint blockade. EBV-infected lymphoma cells express the inhibitory ligand PD-L1 (142). PD-L1 is also found in EBV-associated gastric cancer (143) and T/NK-cell lymphomas (144). Green et al. showed that EBV infection induced PD-L1 expression in classical Hodgkin lymphoma (145). Preclinical work by Ma et al. has shown that inhibition of PD-1 and CTLA-4 dramatically reduces lymphomas induced by EBV in a mouse model (142). The PD1 antibody pembrolizumab has been effective in relapsed/refractory NK cell lymphomas. In their case series, Kwong et al. showed 5 of 7 patients retained a CR after a median follow-up of 6 months after failing l-asparaginase containing therapy (144). Research into the role of checkpoint inhibitors in EBV-mediated malignancy is ongoing (NCT03586024). Combinations of checkpoint inhibitors with lytic induction may prove to be a promising strategy in the future.

## Future Directions

Induction of EBV from latency to the lytic phase of viral replication has become an attractive method for treating EBV mediated malignancy. As discussed and outlined in Table 2, there are a variety of methods for achieving lytic induction. The methods for induction range from traditional chemotherapies to steroids to HDAC inhibitors. The optimal method for inducing lytic activation has not yet been defined. Many of the patients who receive EBV directed therapy have multiple comorbidities, are immunosuppressed, and have already received more traditional cytotoxic chemotherapy-based regimens. Identifying the ideal regimen to induce lytic reactivation and target EBV while reducing the degree of treatment related morbidity and mortality associated with traditional cytotoxic chemotherapy is an active area of research. Combining antiviral targeted approaches with immune based therapies that permit a functional adaptive immune system (or an “off the shelf” allogeneic CTL) may be a potentially rational synergist approach. Perhaps combination of the “kick and kill” approach with checkpoint blockade and/or CTLs (auto or allo) will be a simultaneous or sequential treatment strategy of the future.

## Author Contributions

JD wrote the first draft of the manuscript and contributed to every draft thereafter. CC contributed to later drafts of the manuscript and provided expertise in virology. BH contributed to every iteration of the manuscript and provided expertise in EBV mediated malignancy.

### Conflict of Interest Statement

The authors declare that the research was conducted in the absence of any commercial or financial relationships that could be construed as a potential conflict of interest.
